# Immune reconstitution inflammatory syndrome from Penicillium marneffei in an HIV-infected child: a case report and review of literature

**DOI:** 10.1186/1471-2334-12-28

**Published:** 2012-01-31

**Authors:** Tavitiya Sudjaritruk, Thira Sirisanthana, Virat Sirisanthana

**Affiliations:** 1 Division of Infectious Diseases, Department of Pediatrics, Faculty of Medicine, Chiang Mai University, 50200 Chiang Mai, Thailand; 2 Research Institute for Health Sciences, Chiang Mai University, Chiang Mai, Thailand

## Abstract

**Backgrounds:**

Disseminated *Penicillium marneffei *infection is one of the most common HIV-related opportunistic infections in Southeast Asia. Immune reconstitution inflammatory syndrome (IRIS) is a complication related to antiretroviral therapy (ART)-induced immune restoration. The aim of this report is to present a case of HIV-infected child who developed an unmasking type of IRIS caused by disseminated *P. marneffei *infection after ART initiation.

**Case presentation:**

A 14-year-old Thai HIV-infected girl presented with high-grade fever, multiple painful ulcerated oral lesions, generalized non-pruritic erythrematous skin papules and nodules with central umbilication, and multiple swollen, warm, and tender joints 8 weeks after ART initiation. At that time, her CD4^+ ^cell count was 7.2% or 39 cells/mm^3^. On admission, her repeated CD4^+ ^cell count was 11% or 51 cells/mm^3 ^and her plasma HIV-RNA level was < 50 copies/mL. Her skin biopsy showed necrotizing histiocytic granuloma formation with neutrophilic infiltration in the upper and reticular dermis. Tissue sections stained with hematoxylin and eosin (H&E), periodic acid-Schiff (PAS), and Grocott methenamine silver (GMS) stain revealed numerous intracellular and extracellular, round to oval, elongated, thin-walled yeast cells with central septation. The hemoculture, bone marrow culture, and skin culture revealed no growth of fungus or bacteria. Our patient responded well to intravenous amphotericin B followed by oral itraconazole. She fully recovered after 4-month antifungal treatment without evidence of recurrence of disease.

**Conclusions:**

IRIS from *P. marneffei *in HIV-infected people is rare. Appropriate recognition and properly treatment is important for a good prognosis.

## Background

*Penicillium marneffei *is a dimorphic fungus which can cause a fatal systemic mycosis in human immunodeficiency virus (HIV)-infected patients. This organism is endemic in tropical Asia, especially Thailand, northeastern India, China, Hong Kong, Vietnam, and Taiwan [1-6]. Disseminated *P. marneffei *infection is one of the most common HIV-related opportunistic infections in northern Thailand [5]. The typical manifestations of *P. marneffei *infection in HIV-infected individuals include fever, anemia, weight loss, skin lesions, generalized lymphadenopathy, and hepatomegaly [5,7]. The primary treatment with amphotericin B and itraconazole and secondary prophylaxis with itraconazole are very effective regimens [8]. Patients who do not receive timely and appropriate antifungal treatment have poor outcomes [5]. Immune reconstitution inflammatory syndrome (IRIS) is a complication related to antiretroviral therapy (ART)-induced immune restoration. IRIS manifests as a paradoxical exacerbation of previously treated opportunistic infections (paradoxical or worsening IRIS) or as an unmasking of subclinical, untreated infections (unmasking IRIS) [9-12]. It is a consequence of exaggerated activation of the immune response against infectious organisms [13,14]. In this report, we present a case of HIV-infected child with IRIS from disseminated *P. marneffei *infection.

## Case presentation

A 14-year-old Thai girl presented at a provincial hospital with fever, oral ulcers, disseminated papular lesions and multiple joint pain for 4 weeks. Twelve weeks before this admission, she was diagnosed as a case of perinatal HIV infection after presenting with disseminated herpes zoster infection and *Pneumocystis jirovecii *pneumonia (PJP). At that time, her CD4^+ ^cell count was 7.2% or 39 cells/mm^3^. Plasma HIV-RNA level was not obtained. She was started on GPOvirS30^® ^(a fixed drug combination of stavudine 30 mg, lamivudine 150 mg and nevirapine 200 mg) 1 tablet twice daily and PJP prophylaxis with trimethoprim-sulfamethoxazole. Eight weeks after starting ART she developed fever, multiple oral ulcers, disseminated papular lesions over the face, body, and extremities, and severe pain in many joints. The symptoms did not respond to many kinds of oral antibiotics and she was referred to Chiang Mai University (CMU) Hospital.

**Figure 1:**
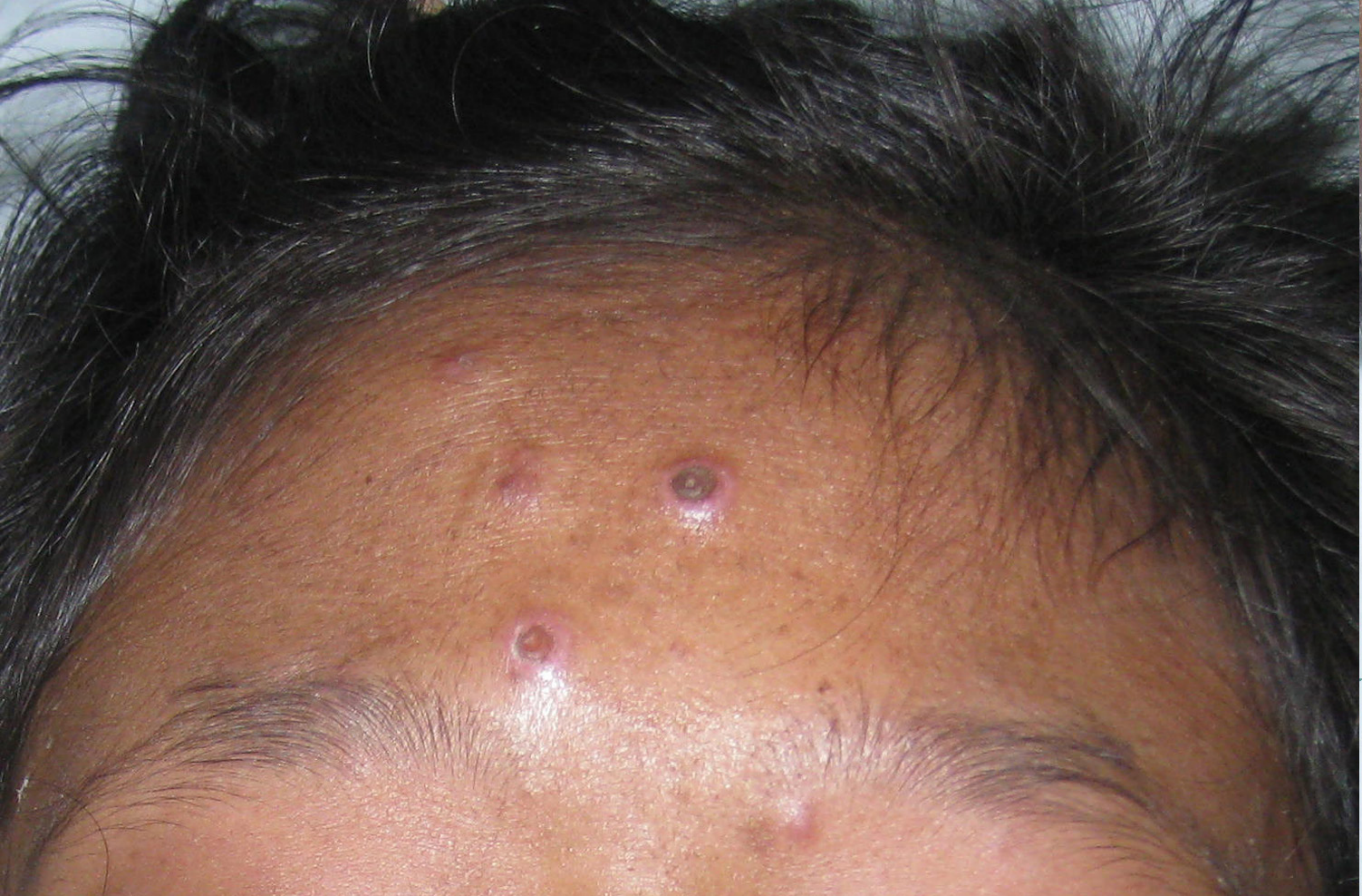
**Cutaneous lesions initially presented as small papules, enlarged to larger papules with central necrotic umbilications**. They were predominantly found on the face and extremities.

**Figure 2:**
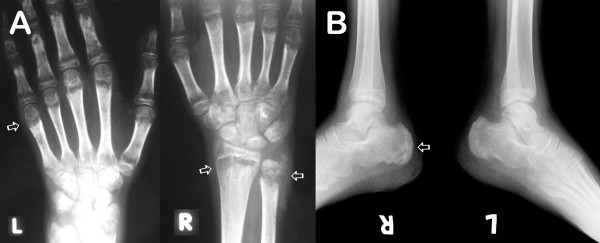
**Radiologic evidences of osteolytic lesions of the extremities**. **a**. Multiple osteolytic lesions are noted along the metaphyseal line of the 2nd to 4th metacarpophalangeal joints (arrow) with pericarticular osteopenia of the wrist and metacarpophalangeal joints. Large osteolytic lesions are also noted at right distal radius and ulnar (arrows). No widening of both wrist joint spaces. Sharp bony cortex of both radius and ulnar. **b**. Multiple osteolytic lesions are noted at right calcaneous (arrow). No widening of both ankle joint spaces. Sharp bony cortex of both tibia and fibula.

**Figure 3:**
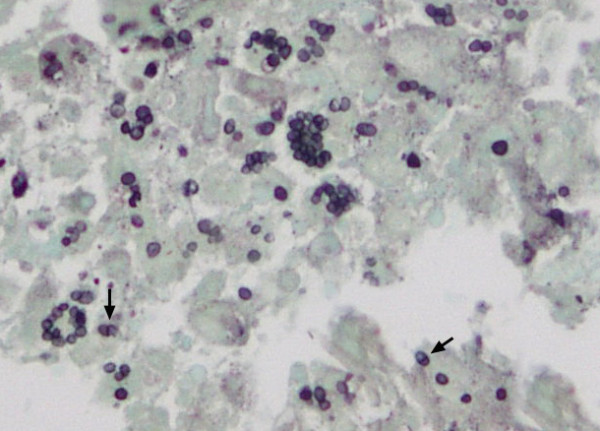
**Photomicrograph of *Penicillium marneffei *(courtesy of Kornkanok Sukapan) in the skin lesion section stained with Grocott Methenamine Silver**. Numerous intracellular and extracellular, round to oval, elongated, thin-walled yeast-like organisms. The characteristic transverse septum (arrows) within the yeast cell is seen. Magnification, × 1000.

Upon admission to CMU Hospital, the physical examination revealed high-grade fever. Multiple painful ulcerated lesions were found on the lip and oral mucosa. Generalized non-pruritic erythrematous papules and nodules with central umbilication were found over the face, body, and extremities (Figure 1). Marked swelling, warmth, and tenderness of many joints, including right shoulder, wrist, metacarpophalangeal, knee, and ankle joints were also noticed. Neither lymphadenopathy nor hepatosplenomegaly were found. Her laboratory results are shown in Table 1. CD4^+ ^cell count was 11% or 51 cells/mm^3^. Plasma HIV-RNA level was < 50 copies/mL. Chest roentgenogram was normal. Roentgenograms of both wrists and ankles showed multiple round radiolucent defects of the bones (Figure 2). Skin biopsy showed necrotizing histiocytic granuloma formation with neutrophilic infiltration in the upper and reticular dermis. Tissue sections from skin biopsy stained with hematoxylin and eosin (H&E), periodic acid-Schiff (PAS), and Grocott methenamine silver (GMS) stain revealed numerous intracellular and extracellular, round to oval, elongated, thin-walled yeast cells with central septation (Figure 3). No organism was observed in the bone marrow aspirate specimen. The hemoculture, bone marrow culture, and skin culture revealed no evidence of *P. marneffei *or other fungus. Blood was also sent for mycobacterial culture with negative results. Serum cryptococcal antigen was negative. The diagnosis of disseminated *P. marneffei *infection from unmasking IRIS was made.

**Table 1:** Laboratory results on the day of admission

Laboratory investigations	Results	Normal value
Hemoglobin (g/dL)	8.0	10.0-15.0

Hematocrit (%)	23.9	36.0-45.0

White blood count (×10^9^/L)	7.6	5-10

Absolute neutrophil count	5.9	2.0-8.0

Absolute lymphocyte count	0.5	0.7-4.4

Platelet (×10^9^/L)	498	100-400

SGOT (U/L)	21	< 35

SGPT (U/L)	10	< 41

LDH (U/L)	161	120-450

ESR (mm/hr)	> 140	0-10

CRP (mg/L)	80.7	0-5.0

**Note: **SGOT indicates Serum glutamic oxaloacetic transaminase; SGPT, Serum glutamic pyruvic transaminase; LDH, Lactate dehydrogenase; ESR, Erythrocyte sedimentation rate; CRP, C-reactive protein

The patient was treated with intravenous amphotericin B at a dosage of 0.7 mg/kg for 2 weeks along with paracetamol and ibuprofen, followed by oral itraconazole at a dosage of 5 mg/kg twice daily orally. She responded well to treatment. Her fever, skin lesions, and joints pain gradually resolved. She was discharged with a plan to complete a 10-week course of oral itraconazole therapy followed by the maintenance therapy with oral itraconazole at a reduced dosage of 5 mg/kg daily. Her skin lesions and joints pain resolved after 4 weeks of antifungal treatment, and itraconazole was discontinued after 4 months of maintenance treatment. After 1 year of therapy, she had gained 4 kg weight without recurrence of *P. marneffei *infection. Her repeated CD4^+ ^cell count had risen to 21.1% or 269 cells/mm^3^. Her plasma HIV RNA level was undetectable (< 50 copies/mL).

## Conclusions

We reported a case of HIV-infected child who developed an unmasking IRIS caused by disseminated *P. marneffei *infection 8 weeks after ART initiation. After treatment with antifungal therapy, amphotericin B followed by itraconazole, she had fully recovery without evidence of recurrence.

IRIS is a manifestation of vigorous immune recovery which usually occurs within a few weeks to months after potent ART initiation in advanced stage HIV-infected patients. This inflammatory reaction is directed against pathogens causing latent or subclinical infection. The majority of patients present with unusual manifestations of opportunistic infections, most often while the number of CD4^+ ^cell count is increasing and/or the plasma HIV RNA level is decreasing [11,12,15,16]. This syndrome can be severe, and results in significant morbidity and occasional mortality. The information about incidence and spectrum of IRIS in HIV-infected children was limited. Puthanakit *et al. *reported an incidence of 19% of IRIS in advanced stage Thai HIV-infected children with the median onset of 4 weeks (range, 2-31) after ART commencement. The three major causative pathogens were *mycobacterial spp*. (43.8%), both *Mycobacterium tuberculosis *(TB) and non-tuberculous mycobacterium, varicella zoster virus (VZV) (21.9%), and herpes simplex virus (HSV) (21.9%) [17]. Recently, Smith *et al. *reported an incidence of 21% of IRIS in South African HIV-infected children at a median of 16 days (range, 7-115 days) post-ART initiation. Bacillus Calmette-Guérin reaction (71%) and TB (35.3%) were the most common conditions in their children [18]. Similarly, Wang *et al. *reported an incidence of 20% of IRIS (19.8 events per 100 person years) in HIV-infected children in Peru with 6.6 weeks (range, 2-32) median time to IRIS. The most common IRIS events were VZV infection (33.3%), HSV labialis (33.3%), and TB infection (22.2%) [19].

Our patient developed symptoms 8 weeks after the initiation of ART, which is a common period for IRIS development. They included fever, multiple painful oral ulcers, disseminated umbilicated papular skin lesions over the face, body and extremities, and multiple swollen, warm, and tender joints which are typical clinical presentations of disseminated *P. marneffei *infection. Tissue sections of the skin biopsy stained with H&E, PAS, and GMS revealed numerous yeast cells of *P. marneffei*, but culture did not yield the organism These signs and symptoms, especially prominent inflammatory articular manifestations, and an excessive inflammation reaction in histopathology of the skin biopsyspecimen demonstrated vigorous immune recovery which acted on unviable *P. marneffei *antigens. Improvement of her immune response was also evidenced by her rising CD4^+ ^cell count and undetectable plasma HIV RNA level. The improved immune response had unmasked a previously quiescent *P. marneffei *infection causing the patient's symptoms.

*P. marneffei *is an important causative organism of opportunistic infection in immunocompromised people, particularly HIV-infected persons who live in or travel to Southeast Asia [4-7,20]. By reviewing the English medical literature, *P. marneffei *had been reported as a causative organism of IRIS in only 4 HIV-infected patients. The first case was reported in 2007 from India [21]. Since then, there have been 2 additional cases reported from the Indian subcontinent [22,23], and 1 case from United Kingdom who had traveled to Thailand [24]. Case histories and the characteristics of these 4 cases are summarized in Table 2. All patients lived in or traveled to an endemic area of *P. marneffei*. Similar with our patient, all except one case (case 1) had evidences of immune recovery during IRIS presentation which occurred within 2-4 weeks after ART initiation. Both kinds of IRIS presentations were reported, 3 as unmasking, and 1 as paradoxical types. All except 1 patient (case 1) had generalized skin and/or mucocutaneous lesions which are the common clinical characteristics of *P. marneffei *infection. The common presenting symptoms were generalized skin and/or mucocutaneous lesions, pyrexia, lymphadenopathy, hepatomegaly and splenomegaly. Our patient also had osteoarticular involvement by clinical and/or radiological findings, similar to case 2 in Table 2. The osteomyelitis was seen in multiple including flat bones, long bones of the extremities and the small bones of hands and feet. The arthritis could involve both large peripheral joints and small joints of the fingers [20,25-27]. These findings were similar to those observed in tuberculosis and systemic mycoses other than penicilliosis [28].

**Table 2:** Immune reconstitution inflammatory syndrome with disseminated *Penicillium marneffei *infection in HIV-infected patients: Literature review

Case	Country reported year	Age (yr)	Sex	Status before ART commencement			Type of ART	Type of IRIS	Time to IRIS onse	Status during IRIS presentation			Method for diagnosis	Treatments	Outcomes
											
				Clinical symptoms	CD4 cell count (cells/mm^3^)	Viral load (copies/mL)				Clinical symptoms	CD4 cell count (cells/mm^3^)	Viral load (copies/mL)			
1	India^24^, 2007	35	M	fever, loss of weight and appetite, hepatosplenomegaly, herpes genitalis	4	NA	d4T, 3TC, NVP	unmasking	4 weeks	afebrile, pallor, mild icterus, cervical and axillary lymphadenopathy, hepatosplenomegaly	NA	NA	axillary LN biopsy-positive, LN culture-positive blood culture -positive,	AmphoB 0.6 MKD for 14 days, followed by itraconazole 400 mg/d for 10 wks, then MT with 200 mg/d	At 10 mo; 20 kg weight gain, decrease size of LN, liver, spleen, CD4 = 224 cells/mm^3^

2	India^25^, 2009	12	M	fever, cough, weight loss, diarrhea, generalized papular umbilicated lesion, oral and esophageal candidiasis	11	NA	d4T, 3TC, EFV	paradoxical	4 weeks	fever, severe arthritis, exacerbration of skin lesions, generalised lymphadenopathy	172 (wk 4)	NA	blood culture-positive	NA	NA

3	India^26^, 2010	28	M	fever, cough, loss of weight, diarrhea, oral candidiasis	47	NA	d4T, 3TC, NVP	unmasking	2 weeks	multiple erythrematous, scaly, papules and nodules with central necrosis on face extremities, scortum	160 (wk 2)	NA	skin biopsy-positive, skin culture-positive, blood culture-negative	AmphoB 0.6 MKD only 1 dose, then itraconazole 400 mg/d for 2 mo, then MT with 200 mg/d	At 2 mo; 14 kg weight gain, skin lesions disappear

4	UK (traveled to Thailand)^27^, 2010	39	M	fever, loss of weight and appetite, PJP, molluscum contangiosum on face	72	38000000	TDF, FTC, EFV	unmasking	4 weeks	multiple facial lesions, disseminated non-pruritic nodules, no hepatosplenomegaly	273 (wk 8)	3 log drop (wk 4)	pus culture-positive	AmphoB 0.6 MKD for 14 days, followed by itraconazole 600 mg/d for 10 wks, then MT with 200 mg/d	At 2 mo; skin lesions regress At 28 mo; CD4 = 375 cells/mm^3^, VL < 50 copies/mL

5	Thailand, 2011 (Ours)	14	F	fever, loss of weight and appetite, PJP, herpes zoster on trunk	39	NA	d4T, 3TC, NVP	unmasking	8 weeks	fever, severe osteoarthritis, disseminated non-pruritic papules and nodules with central necrosis, oral ulcer, no lymphadenopathy, no hepatosplenomegaly	51 (wk 14)	< 50 (wk 14)	skin biopsy-positive, skin culture-negative, blood culture-negative	AmphoB 0.7 MKD for 14 days then Itraconazole 5 MK twice daily for 10 weeks, then MT with 5 MKD for 4 months	At 12 mo; 4 kg weight gain, CD4 = 269 cells/mm^3^, VL < 50 copies/mL

**Note: **M indicates male; d4T, stavudine; 3TC, lamivudine; TDF, tenofovir; FTC, emtricitabine, EFV, efavirenz; NVP, nevirapine; LN, lymph node; AmphoB, Amphotericin B deoxycholate; MKD, mg/kg/day; MT, maintenance; VL, plasma HIV RNA level; NA, not available

Diagnosis of infection by *P. marneffei *is usually made by identifying the fungus in clinical specimens by microscopy and culture. In addition, *P. marneffei *can be seen in histopathological sections stained with H&E, GMS, or PAS stain, which typically appears as unicellular round to oval yeast cells with transverse septum in macrophage or histiocyte [29]. This finding is unique to infection with *P. marneffei*. All 4 previously reported cases had evidences of the fungus in the clinical specimens, including blood, skin biopsies, and lymph node biopsies and culture [21-24]. However, in our reported case, we found the evidences of organism in histopathological sections with special stains but could not identify fungus by culture of the clinical specimens. This might be due to the fact that the organism was already killed by the immune system of the patient.

Treatment of disseminated *P. marneffei *infection is well described [30]. Intravenous amphotericin B for 2 weeks followed by oral itraconazole for 10 weeks is recommended. Although itraconazole maintenance treatment was shown to prevent relapse of penicilliosis when the patients had CD4 cell count of 100 cells/μl or greater for at least 6 months after HAART [31], our patient who unintentionally discontinued maintenance treatment after 4-month therapy without knowing the CD4 cell count did not have relapse. Details of treatments and outcomes were available in 3 of 4 previously reported cases (Table 2). Two cases (case 1, 4) were treated with intravenous amphotericin B for 2 weeks, followed by oral itraconazole for 10 weeks. The other case (case 3) received single dose of intravenous amphotericin B because the patient refused to stay in the hospital. Therefore, he was treated by only itraconazole orally for 8 weeks. All 3 cases were continued on maintenance treatment with oral itraconazole along with the ART. Their symptoms improved markedly after 2-10 months of the therapy.

In summary, IRIS is not a rare condition, especially in the ART era. IRIS caused by *P. marneffei *infection will be increasingly recognized in the endemic area of the fungus. It requires appropriate recognition and proper treatment. The clinicians' awareness is crucial to ensure a good prognosis.

## Consent

Written informed consent was obtained from the parents of the patient for publication of this case report and any accompanying images. A copy of the written consent is available for review by the Editor-in-Chief of this journal.

## Competing interests

The authors declare that they have no competing interests.

## Authors' contributions

All authors contributed to this work. TAS and VS were involved in the direct clinical care (diagnosis, decision making, and treatment) of the reported patient. Both provided the corresponding figures. All authors involved in the preparation of the manuscript. All authors read and approved the final version of the manuscript.

## Funding

None.

## Ethical approval

This study was approved by Ethics Committee of Faculty of Medicine, Chiang Mai University, Chiang Mai, Thailand.

## Pre-publication history

The pre-publication history for this paper can be accessed here:

http://www.biomedcentral.com/1471-2334/12/28/prepub
